# Identification of functional murine mitochondrial formyl peptides and their effects on myeloid‐derived suppressor cell generation

**DOI:** 10.1002/2211-5463.70209

**Published:** 2026-02-11

**Authors:** Miyako Ozawa, Saori Kagoshima, Akira Yamada

**Affiliations:** ^1^ Tumor Immunology Division, Research Center for Innovative Cancer Therapy Kurume University Japan

**Keywords:** formyl, MDSCs, mitochondria, mouse, peptide, receptors

## Abstract

N‐formyl peptides are cleavage products of bacterial and mitochondrial proteins, which effects on neutrophil activation have been extensively studied. Mitochondrial formyl peptides are also known to play a role in tumor immunity, but the molecular mechanism underlying this role remains largely unexplored. Our previous work suggested that tumor‐derived mitochondrial formyl peptides promote tumor growth by enhancing myeloid‐derived suppressor cell (MDSC)‐mediated cytotoxic T‐lymphocyte suppression. Therefore, we hypothesized that tumor‐derived mitochondrial formyl peptides act directly on bone marrow cells to promote MDSC generation. In this study, we tested this hypothesis by first identifying five functional murine mitochondrial formyl peptides that induced intracellular Ca^2+^ flux and chemotaxis, and then assessing their effects on the *in vitro* generation of MDSCs from murine bone marrow cells cultured with GM‐CSF and IL‐6. Addition of mitochondrial formyl peptides resulted in a 5–10% increase in polymorphonuclear‐MDSCs along with a corresponding decrease in monocyte‐MDSCs. These results indicate that tumor‐derived mitochondrial formyl peptides directly influence MDSC generation and corroborate findings from prior *in vivo* tumor transplantation models.

## Graphical abstract

We first identified functional murine mitochondrial N‐formyl peptides (MT‐FPs) and investigated their effects on the *in vitro* myeloid‐derived suppressor cell (MDSC) generation from bone marrow cells. We demonstrated that MT‐FPs acted directly on bone marrow cells to promote MDSC generation and modulated the polymorphonuclear (PMN)‐MDSC/monocyte (M)‐MDSC balance. These results corroborate findings from prior *in vivo* tumor transplantation models.

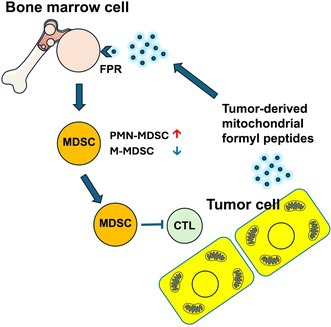

**Abbreviations**
CAR‐T: chimeric antigen receptor T‐cell
CFSE: carboxyfluorescein diacetate succinimidyl ester
DAMP: damage‐associated molecular pattern
FPR: formyl peptide receptor
HMGB1: high‐mobility group box 1
iCa^2+^
: intracellular Ca^2+^
Lm‐FP: *Listeria monocytogenes* derived N‐formyl peptide
MDSC: myeloid‐derived suppressor cell
M‐MDSC: monocyte‐MDSC
mMT‐FP: murine mitochondrial formyl peptide
MTFMT: mitochondrial methionyl‐tRNA formyltransferase
MT‐FP: mitochondrial formyl peptide
PMN‐MDSC: polymorphonuclear‐MDSC
TLR: toll‐like receptor
TME: tumor microenvironment

Recent advances in cancer immunology have allowed immunotherapies, such as immune checkpoint inhibitors and chimeric antigen receptor T‐cell (CAR‐T) therapies, to become major therapeutic modalities against cancers, including blood tumors. However, clinical outcomes remain limited in many cases [1, 2]. Tumor microenvironments (TMEs) consist of various immune‐related cells, including immunosuppressive cells such as regulatory T cells, myeloid‐derived suppressor cells (MDSCs), and tumor‐associated type 2 macrophages, which often hinder immunity against cancer cells [3, 4]. Damage‐associated molecular patterns (DAMPs) derived from tumor cells are among the inducers of these cells in the TME [3, 4]. Several DAMP molecules act via Toll‐like receptors (TLRs) [5, 6]. We previously investigated the role of high‐mobility group box 1 (HMGB1), a representative TLR‐reactive DAMP molecule, in tumor immunity [7, 8]. We also investigated the roles of mitochondrial formyl peptides (MT‐FPs), a representative example of TLR‐independent DAMPs, in tumor immunity [9]. During the initiation of protein synthesis, formyl‐methionine is used as the N‐terminal amino acid in mitochondria as well as in bacteria; by contrast, it is absent from nuclear‐encoded protein synthesis in eukaryotic cells [10, 11]. Formyl peptide receptors (FPRs) belong to the G‐protein–coupled receptor family and contain seven transmembrane‐spanning regions with an extracellular N terminus [12, 13, 14, 15]. At least two functional FPRs, FPR‐1 and FPR‐2, are known to be expressed in mice, and signaling through these receptors induces Ca^2+^ flux in various types of cells and chemotaxis of neutrophils [12, 13, 14, 15]. Formylation of methionine is catalyzed by mitochondrial methionyl‐tRNA formyltransferase (MTFMT) in eukaryotic cells [10, 11]. We established MTFMT‐knockout B16F10 and CT26 tumor cells and investigated their *in vivo* growth following subcutaneous transplantation into syngeneic mice [9]. MTFMT‐knockout tumor cell growth was suppressed by CTL‐mediated antitumor immunity. Intratumor (i.t.) injection of a murine MT‐FP mixture enhanced tumor growth in the MTFMT‐knockout cells *in vivo*. Decreased infiltration of MDSCs, particularly of polymorphonuclear (PMN)‐MDSCs, into the tumor tissues was observed in the MTFMT‐knockout tumors, and i.t. injection of the murine MT‐FP mixture enhanced the MDSC infiltration into the tumor tissues of both the MTFMT‐knockout and wild‐type tumor cells, suggesting that tumor‐derived MT‐FPs enhanced MDSC induction [9]. We hypothesized that tumor‐derived MT‐FPs act directory bone marrow cells to promote MDSC generation. To confirm this hypothesis, we first identified functional murine MT‐FPs that activate bone marrow neutrophils and investigated their effects on the *in vitro* generation of MDSCs from murine bone marrow cells.

## Materials and methods

### Mice

Seven‐week‐old female C57BL/6J (B6) mice were purchased from CLEA Japan (Tokyo, Japan) and housed under specific pathogen‐free conditions at the Institute for Disease Modeling, Kurume University School of Medicine. All animal experimental protocols were approved by the Institutional Animal Care and Use Committee of Kurume University (approval no. 2019‐030) and conducted in accordance with national guidelines on the care and use of laboratory animals. To obtain bone marrow cells, mice were euthanized by cervical dislocation.

### Peptides

The murine mitochondrial N‐formyl peptides (mMT‐FPs) used in this study are shown in Table 1. These peptides were custom‐synthesized and purchased from Genscript (Piscataway, NJ, USA). Additional peptides included fMIVTLF, a *Listeria monocytogenes*–derived N‐formyl peptide (Lm‐FP) from Bio‐Synthesis (Lewisville, TX, USA); fMIFL, a *Staphylococcus aureus*–derived N‐formyl peptide from Anygen (Gwangju, Korea); WKYMVm from Abcam (Cambridge, UK); Boc‐FLFLF (Boc 2) from MedChemExpress (Monmouth Junction, NJ, USA); and WRWWWW (WRW4) from TOCRIS Bioscience (Bristol, UK). All peptides were dissolved in dimethyl sulfoxide (DMSO; FUJIFILM Wako Pure Chemical, Tokyo, Japan) at 10 mg·mL^−1^ unless otherwise specified and diluted appropriately for experiments.

**Table 1:** Murine mitochondrial N‐formyl peptides used in this study.

Peptide name	Sequence	aa length	Parental protein name	NCBI accession
mMT‐ATP8	formyl‐MPQLDT	6	ATP synthase F0 subunit 8	NP_904332.1
mMT‐ATP6	formyl‐MNENLF	6	ATP synthase F0 subunit 6	NP_904333.1
mMT‐CO1	formyl‐MFINRW	6	cytochrome c oxidase subunit I	NP_904330.1
mMT‐CO2	formyl‐MAYPFQ	6	cytochrome c oxidase subunit II	NP_904331.1
mMT‐CO3	formyl‐MTHQTH	6	cytochrome c oxidase subunit III	NP_904334.1
mMT‐CYB	formyl‐MTNMRK	6	cytochrome b	NP_904340.1
mMT‐ND1	formyl‐MFFINI	6	NADH dehydrogenase subunit 1	NP_904328.1
mMT‐ND2	formyl‐MNPITL	6	NADH dehydrogenase subunit 2	NP_904329.1
mMT‐ND3	formyl‐MNLYTV	6	NADH dehydrogenase subunit 3	NP_904335.1
mMT‐ND4L	formyl‐MPSTFF	6	NADH dehydrogenase subunit 4 L	NP_904336.1
mMT‐ND4	formyl‐MLKIIL	6	NADH dehydrogenase subunit 4	NP_904337.1
mMT‐ND5	formyl‐MNIFTT	6	NADH dehydrogenase subunit 5	NP_904338.1
mMT‐ND6	formyl‐MNNYIF	6	NADH dehydrogenase subunit 6	NP_904339.1

### Preparation of bone marrow cells

Bone marrow cells were aseptically harvested from the femurs and tibias of B6 mice. Erythrocytes were lysed by hypotonic treatment with 0.2% NaCl for 30 s at room temperature. Cells were then washed and suspended in RPMI 1640 medium (Nacalai Tesque, Kyoto, Japan) supplemented with 10 mm HEPES, 10% FCS (Cytiva Marlborough, MA, USA), and antibiotics unless otherwise indicated.

### Measurement of intracellular Ca^2+^ mobilization

Bone marrow cells were labeled with Fluo 4, a Ca^2+^ indicator, using the Calcium Kit‐Fluo 4 (Dojindo, Kumamoto, Japan) according to the manufacturer's protocol. Briefly, cells (1 × 10^6^ cells·mL^−1^) suspended in 10% FCS‐RPMI 1640 medium were incubated with 5 μm Fluo 4‐AM at 37 °C for 1 h. After washing, cells were resuspended in the same medium at 0.5 × 10^6^ cells·mL^−1^. Aliquots of 0.15 mL were analyzed for intracellular Ca^2+^ (iCa^2+^) levels using a FACS CantoII flow cytometer (BD Biosciences, San Jose, CA, USA). Baseline iCa^2+^ levels were recorded for 20–30 s before mMT‐FPs or other stimulants were added, followed by iCa^2+^ measurements for an additional 60 s. The percentage of responding cells was calculated from histogram data collected during the 30‐s poststimulation. In some experiments, cells were pre‐incubated with 10 μm of antagonists at 37 °C for 5 min.

### Chemotaxis assay

Bone marrow cells were suspended in X‐VIVO 15 (Lonza, Basel, Switzerland) supplemented with 10 mM HEPES at a concentration of 1 × 10^7^ cells·mL^−1^. The chemotaxis assay was performed using a 6.5 mm Transwell^®^ plate with a 3.0 μm pore‐size membrane insert (Corning, Corning, NY, USA). A total of 0.5 mL of 10 mm HEPES‐X‐VIVO medium was added to each outer well, and 0.1 mL of the bone marrow cell suspension (1 × 10^6^ cells/0.1 mL) was added to the insert well. mMT‐FPs or control peptides were added to the outer wells. The insert was then placed into the outer wells and incubated at 37 °C in a 5% CO_2_ incubator for 45 min. Cells that migrated into the outer wells were counted using a FACS Canto II flow cytometer. In some experiments, cells were pre‐incubated with 10 μm antagonists at 37 °C for 5 min.

### *In vitro* generation and identification of MDSCs

Bone marrow cells were cultured with 20 ng·mL^−1^ GM‐CSF (PeproTech, Rocky Hill, NJ, USA) and 20 ng·mL^−1^ IL‐6 (Pepro Tech) in RPMI 1640 medium (10% FCS) supplemented with 1 mm sodium pyruvate, 50 μg·mL^−1^ gentamicin, and 50 μm 2‐mercaptoethanol at a density of 0.45 × 10^6^ cells per 1.5 mL per well in a 12‐well plate at 37 °C for 4 days. Adherent cells were harvested using 1 mm EDTA and cell scrapers. Cells were then treated with anti‐CD16/CD32 (BioLegend, San Diego, CA, USA) to block Fc receptors, stained with Alexa Fluor 488‐anti‐CD11b, PerCP/Cy5.5‐anti‐Ly6C, and APC/Cy7‐anti‐Ly6G antibodies (BioLegend), and analyzed using the FACS Canto II flow cytometer. CD11b^+^ Ly6C^dull^ Ly6G^+^ cells and CD11b^+^ Ly6C^bright^ Ly6G^−^ cells were defined as PMN‐MDSCs and monocyte‐MDSCs (M‐MDSCs), respectively [16].

### Functional analysis of MDSCs

The immunosuppressive effect of MDSCs on T‐cell activation was analyzed using carboxyfluorescein diacetate succinimidyl ester (CFSE) cell proliferation assay. Spleen cells were obtained from B6 mice and suspended in PBS at a concentration of 3 × 10^6^ cells·mL^−1^. The cells were labeled with 0.1 μm CFSE for 15 min at 37 °C using the CellTrace™ CFSE Cell Proliferation Kit (Thermo Fisher Scientific, Tokyo, Japan) [17]. The CFSE‐labeled cells were suspended in 10% FCS‐RPMI 1640 at a concentration of 6.7 × 10^6^ cells·mL^−1^. Each 180 μL (1.2 × 10^6^ cells) aliquot of the CFSE‐labeled spleen cells and 155 μL of MDSCs or medium at different spleen cell/MDSC ratios was placed into a well of a 48‐well plate and stimulated with 25 μL of Dynabeads mouse T‐cell activator CD3/CD28 (Life Technologies, Carlsbad, CA, USA) for 3 days at 37 °C in a 5% CO_2_ atmosphere. After culture, cells were harvested, stained using APC/Cy7‐anti‐CD4 or APC/Cy7‐anti‐CD8 antibodies (BioLegend), and analyzed using the FACS Canto II flow cytometer.

### Flow cytometric analysis of chemokine receptors

PE‐conjugated anti‐CCR1 (CD191), ‐CCR2 (CD192), ‐CCR5 (CD195), ‐CXCR2 (CD182) (BioLegend), and ‐CXCR1 (CD181) (BD Biosciences) were used for staining and analyzed using the FACS Canto II flow cytometer. PE‐conjugated rat IgG2a, IgG2b, and Armenian hamster IgG (BioLegend) were also used as isotype controls.

### Statistical analysis

Differences between the two groups were analyzed by one‐way analysis of variance (ANOVA) followed by Tukey's honestly significant difference (HSD) test for post hoc comparisons. The data were analyzed by paired *t*‐tests (Fig. 5C). A *P*‐value < 0.05 was considered statistically significant. Statistical analyses were performed using jmp pro version 17 software (SAS Institute, Cary, NC, USA).

## Results

### Effect of mMT‐FPs on intracellular Ca^2+^ levels in bone marrow neutrophils

**Fig. 1:**
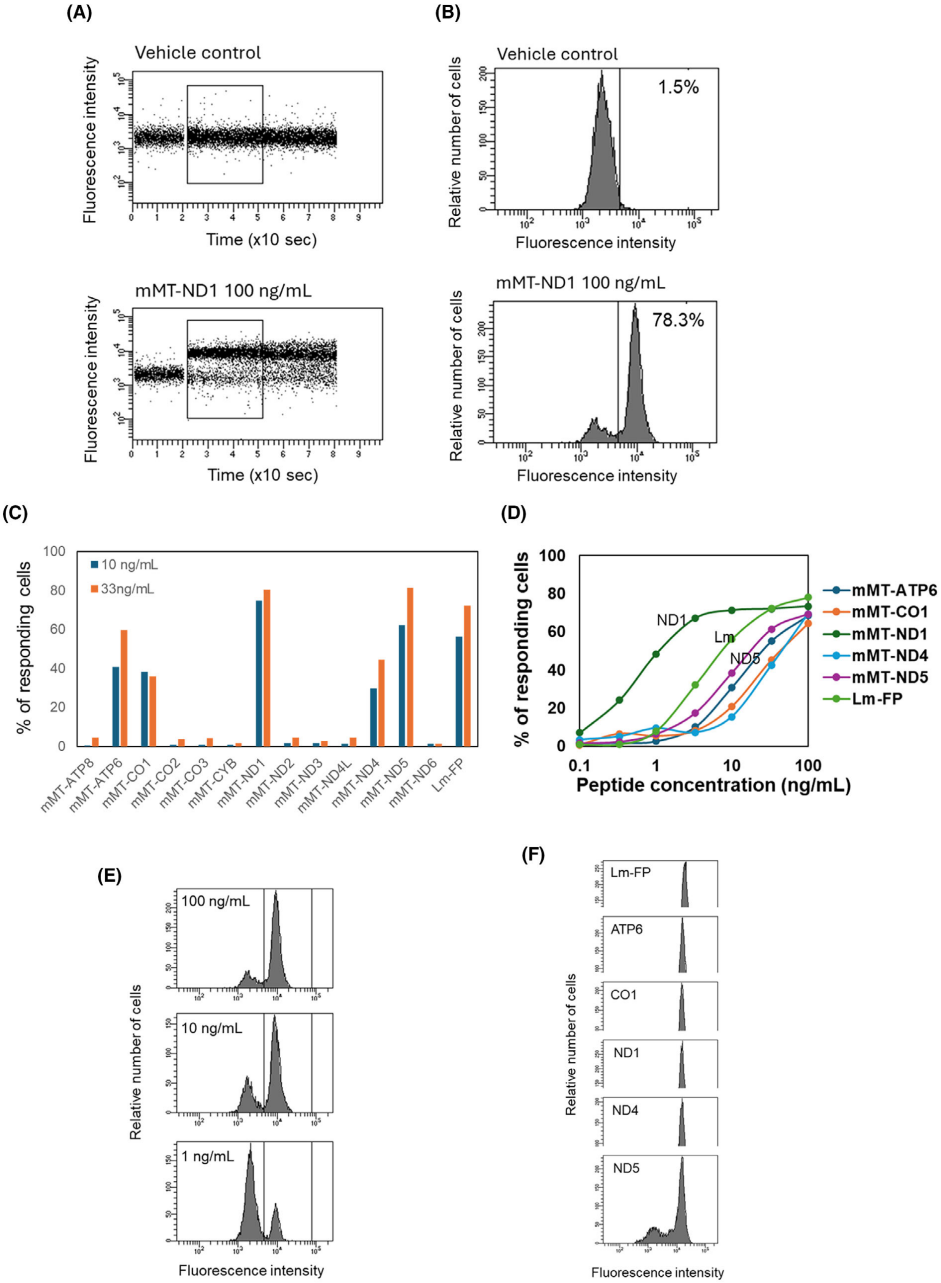
Murine bone marrow cells were labeled with Fluo 4‐AM, and intracellular Ca^2+^ (iCa^2+^) levels were measured using a FACS CantoII flow cytometer. (A) Initial iCa^2+^ levels were measured during the first 20–30 s. mMT‐FPs or other stimulants were then added, and iCa^2+^ levels were measured for the subsequent 60 s. (B) Percentages of responding cells were calculated from the histograms of iCa^2+^ levels during the 30 s period after stimulation. (C) Percentages of cells responding to each mMT‐FP and to the control Lm‐FP at concentrations of 10 and 33 ng·mL^−1^. (D) Dose–response curves of the five mMT‐FPs and Lm‐FP. (E) Representative histograms of the three doses (100, 10, 1 ng·mL^−1^) of mMT‐ND1 peptide. (F) iCa^2+^ levels in responding cells for the five peptides and Lm‐FP. Representative results from three independent experiments.

The binding of formyl peptides to their receptors on neutrophils and subsequent intracellular Ca^2+^ (iCa^2+^) flux levels varied among the different sequences of peptides, including human MT‐FPs [18, 19]. We therefore assessed the ability of the 13 mMT‐FPs to trigger iCa^2+^ flux in murine bone marrow neutrophils after stimulation. The *Listeria monocytogenes*–derived peptide (Lm‐FP, formyl‐MIVTLF) served as a positive control [18]. Fluo 4‐labeled murine bone marrow cells were stimulated with FPs, and iCa^2+^ levels were measured by flow cytometry. A representative result from three independent flow cytometric analyses is shown in Fig. 1A. iCa^2+^ levels increased rapidly following stimulation with the mMT‐ND1 peptide but did not increase following vehicle control treatment. Histograms in Fig. 1B display the fluorescence intensity, relative cell counts, and percentages of cells responding within 30 s of peptide stimulation. The proportions of responding cells for each mMT‐FP and the control Lm‐FP at 10 and 33 ng·mL^−1^ are shown in Fig. 1C. Of the 13 mMT‐FPs tested, 5 (mMT‐ATP6, mMT‐CO1, mMT‐ND1, mMT‐ND4, and mMT‐ND5) elicited increased iCa^2+^ levels, while the remaining 8 showed no such effect at either concentration. Dose–response curves for the five active mMT‐FPs and Lm‐FP are shown in Fig. 1D. These results suggest that mMT‐ND1 has the highest capacity for activating bone marrow neutrophils via iCa^2+^ flux, followed by mMT‐ND5, mMT‐ND4, mMT‐ATP6, and mMT‐CO1 in that order. Among the different peptide doses, the percentages of the responding cells varied; however, the iCa^2+^ levels of the responding cells were almost the same. Representative histograms of the three doses of mMT‐ND1 peptide are shown in Fig. 1E. Similarly, the iCa^2+^ levels among responding cells for the five peptides and Lm‐FP were nearly the same (Fig. 1F).

### Effect of mMT‐FPs on chemotaxis of bone marrow neutrophils

**Fig 2:**
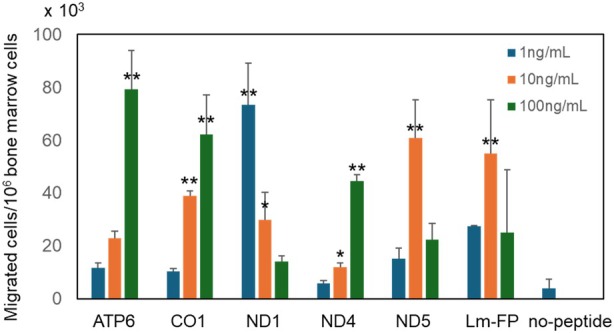
A chemotaxis assay was performed using a 6.5 mm Transwell® plate with a 3.0 μm pore‐size membrane insert. Bone marrow cells (1 × 10^6^ cells/0.1 mL) were added to the insert well, and mMT‐FPs or other peptides were added to the outer wells. Migrated cell numbers after 45 min of incubation with each peptide at three doses (1, 10, 100 ng·mL^−1^) are shown. Differences between the no‐peptide control and peptide groups were analyzed by one‐way ANOVA followed by Tukey's HSD test for post‐hoc comparisons. The error bars indicate SD. *P < 0.05, **P < 0.005. Representative results from three independent experiments.

We analyzed the chemotactic activity of the five mMT‐FPs and the positive control Lm‐FP in bone marrow neutrophils. Migrated cell numbers after 45 min of incubation with each peptide at three concentrations (1, 10, 100 ng·mL^−1^) are shown in Fig. 2. Dose‐dependent activity was observed for mMT‐ATP6, mMT‐CO1, and mMT‐ND4 in neutrophil chemotaxis assays. In contrast, mMT‐ND1 showed the highest activity at the lowest concentration (1 ng·mL^−1^). Both mMT‐ND5 and control Lm‐FP showed bell‐shaped dose–response curves.

### Effect of FPR antagonists on mMT‐FP induced iCa^2^ ^+^ flux and chemotaxis of bone marrow neutrophils

**Fig. 3:**
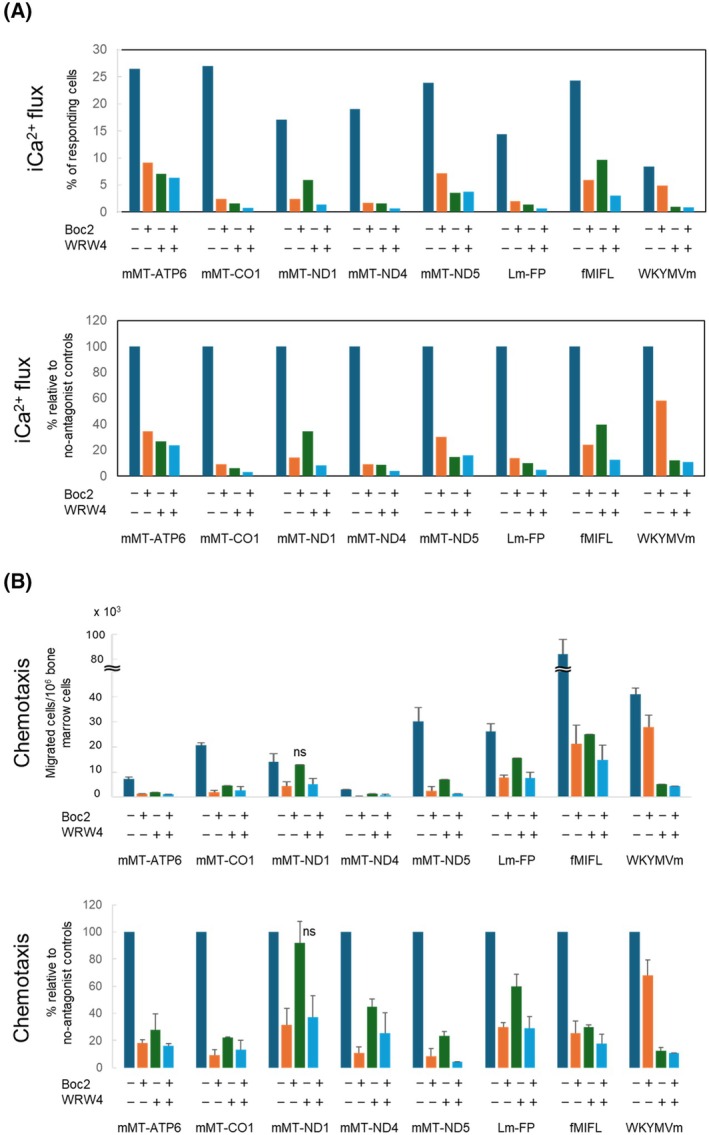
Boc2 and WRW4 were used as FPR‐1 and FPR‐2 antagonists, respectively. *S. aureus*–derived formyl‐MIFL (fMIFL) and WKYMVm were also used as an FPR‐1 agonist and an FPR‐1/FPR‐2 agonist, respectively. (A) Effects of the antagonists on the iCa^2+^ flux induced by the five mMT‐FPs and control peptides were analyzed. The upper panel shows the percentage of responding cells, and the lower panel shows the percentage relative to the no‐antagonist controls. (B) Effects of the antagonists on the chemotaxis of bone marrow neutrophils induced by the five mMT‐FPs and the control peptides were also analyzed. The upper panel shows the number of migrated cells per 10^6^ bone marrow cells, and the lower panel shows the percentage relative to the no–antagonist controls. Differences between the no‐antagonist controls and antagonist groups were analyzed by one‐way ANOVA followed by Tukey's HSD test for post‐hoc comparisons. The error bars indicate SD. n.s., not significant.

Boc2 and WRW4 were used as FPR‐1 and FPR‐2 antagonists, respectively [20]. We also used the *S. aureus*‐derived formyl‐MIFL (fMIFL) as an FPR‐1 agonist and WKYMVm as an FPR‐1/FPR‐2 agonist, in addition to the control Lm‐FP [18]. The effects of these antagonists on iCa^2+^ flux induced by the five mMT‐FPs, Lm‐FP, and the agonists were analyzed (Fig. 3A). Both Boc2 and WRW4 inhibited fMIFL‐ and WKYMVm‐induced responses, with preferential inhibition by Boc2 on the fMIFL‐induced response and by WRW4 on the WKYMVm‐induced response. These results suggested that the specificity of Boc2 and WRW4 was not absolute. Boc2 and WRW4 also inhibited the iCa^2+^ flux induced by the five mMT‐FPs, with preferential inhibition of mMT‐ND1–induced responses by Boc2 and of mMT‐ND5–induced responses by WRW4.

We also analyzed the effects of these antagonists on bone marrow neutrophil chemotaxis induced by the five mMT‐FPs and control peptides (Fig. 3B). WRW4 selectively inhibited WKYMVm‐induced chemotaxis, whereas Boc2 and WRW4 equally inhibited fMIFL‐induced chemotaxis. Both Boc2 and WRW4 inhibited chemotaxis induced by the five mMT‐FPs, except for the mMT‐ND1‐induced response, which Boc2 inhibited alone, as WRW4 had no effect. Preferential inhibition by Boc2 was observed for mMT‐ND4‐ and mMT‐ND5‐induced chemotaxis.

### Effect of FPR agonist‐induced desensitization on mMT‐FP induced iCa^2^ ^+^ flux of bone marrow neutrophils

**Fig. 4:**
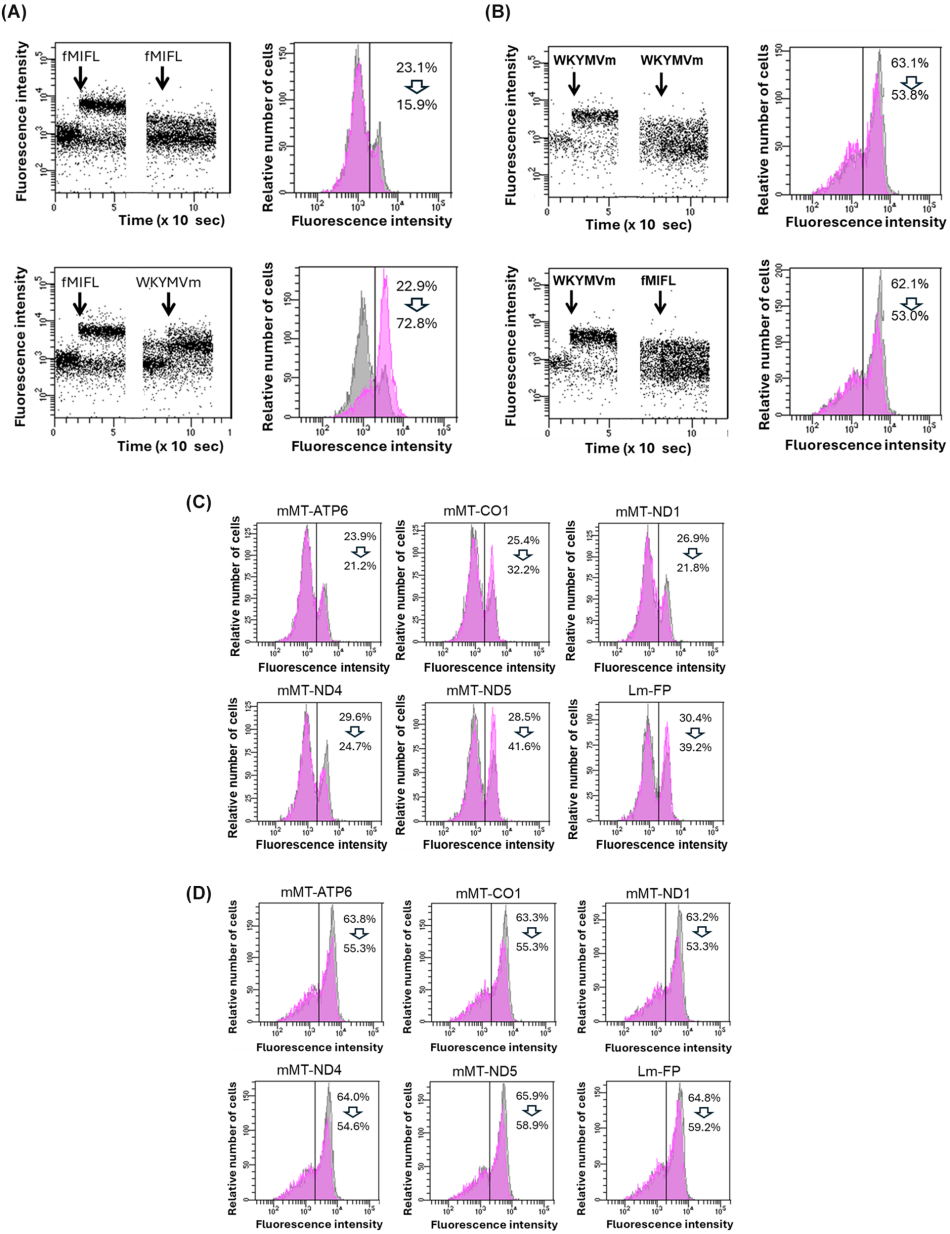
Effects of FPR agonist–induced desensitization on mMT‐FP–induced iCa^2+^ flux in bone marrow neutrophils were examined. Pre‐stimulation of FPR‐1 with fMIFL inhibited subsequent fMIFL‐induced responses but not the WKYMVm‐induced response (A). Pre‐stimulation of FPR‐1/−2 with WKYMVm inhibited both fMIFL‐ and WKYMVm‐induced responses (B). The iCa^2+^ flux responses to the five mMT‐FP and control Lm‐FP following pre‐stimulation with fMIFL (C) or WKYMVm (D). Values of baseline and post‐stimulation with agonists or FPs are shown in the histogram boxes. Representative results from three independent experiments.

FPR‐1 agonist fMIFL and FPR‐1/FPR‐2 agonist WKYMVm were used to desensitize FPRs in bone marrow neutrophils, and subsequent responses to mMT‐FPs were examined using iCa^2+^ flux assay. Prestimulation of FPR‐1 with fMIFL inhibited the subsequent fMIFL‐induced response but not the WKYMVm‐induced response (Fig. 4A). Prestimulation of FPR‐1/−2 with WKYMVm inhibited both the fMIFL‐ and WKYMVm‐induced responses (Fig. 4B). Under these conditions, prestimulation with fMIFL induced unresponsiveness to mMT‐ATP6, mMT‐ND1, and mMT‐ND4. The responses to mMT‐CO1, mMT‐ND5, and Lm‐FP were partially inhibited following fMIFL prestimulation, and the cells still responded to mMT‐CO1, mMT‐ND5, and Lm‐FP (Fig. 4C). In contrast, prestimulation with WKYMVm completely inhibited all five mMT‐FP‐ and Lm‐FP‐induced responses (Fig. 4D). These results suggest that mMT‐CO1, mMT‐ND5, and Lm‐FP activate both FPR‐1 and FPR‐2, while mMT‐ATP6, mMT‐ND1, and mMT‐ND4 activate FPR‐1 specifically in bone marrow neutrophils. Representative results from three independent experiments are shown.

### Effect of mMT‐FPs on *in vitro* generation of MDSCs

**Fig. 5:**
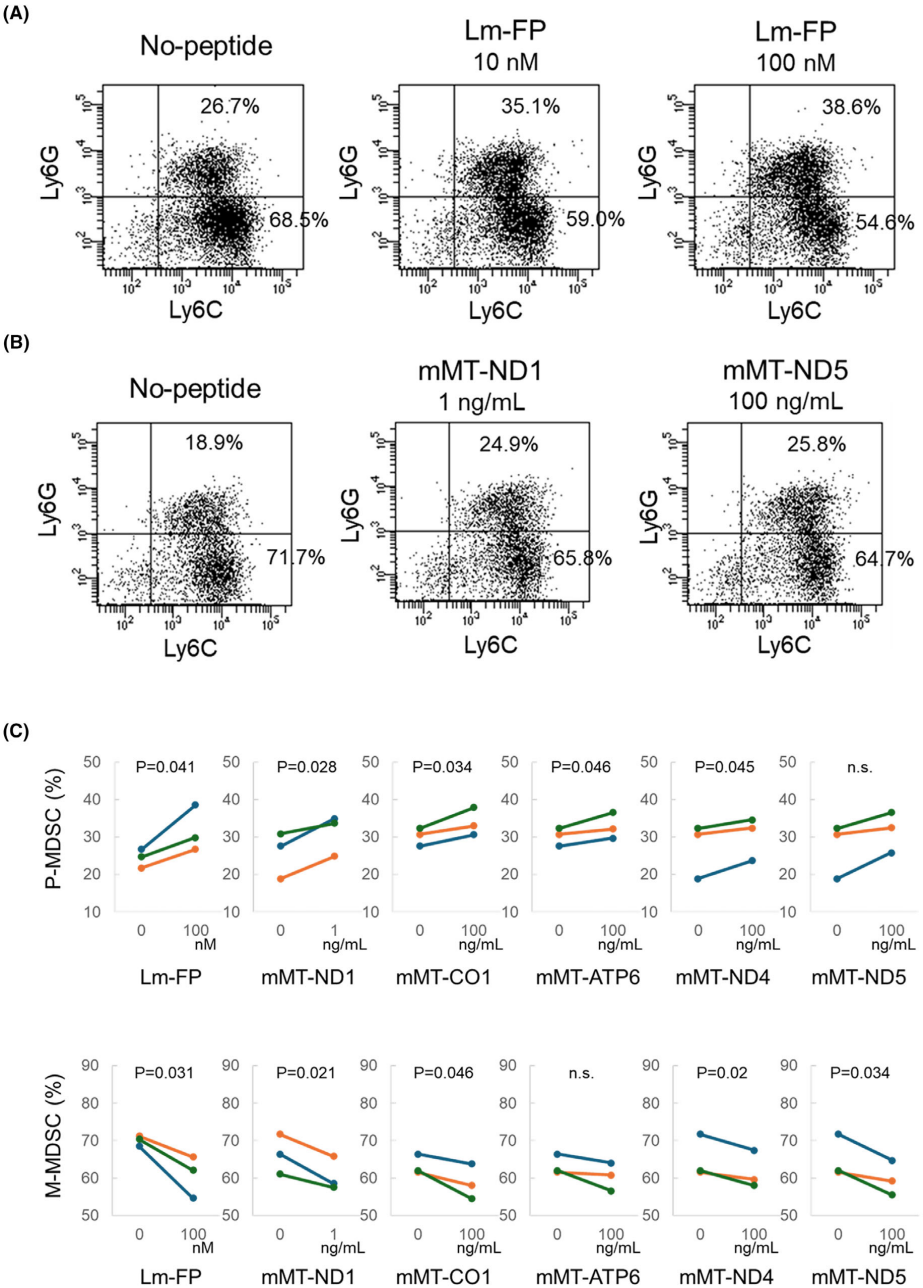
Bone marrow cells were cultured for 4 days in the presence of GM‐CSF and IL‐6. The cells were harvested after the culture period, stained with Alexa Fluor 488‐anti‐CD11b, PerCP/Cy5.5‐anti‐Ly6C, and APC/Cy7‐anti‐Ly6G antibodies and analyzed by flow cytometry. CD11b^+^ cells were gated, and the expression of Ly6C and Ly6G was analyzed. (A) Lm‐FP (10 and 100 nm) was added to the bone marrow culture. Representative results are displayed. The percentages of Ly6C^+^Ly6G^+^ (mainly PMN‐MDSCs) and Ly6C^+^Ly6G^−^ (mainly M‐MDSCs) are also shown. (B) Representative results for mMT‐FPs. (C) Proportions of PMN‐MDSCs (Ly6C^+^Ly6G^+^) and M‐MDSCs (Ly6C^+^Ly6G^−^) in the FP‐treated groups and the corresponding no‐peptide controls in each experiment. The data were analyzed by paired *t*‐tests. n.s., not significant.

We used an *in vitro* system to generate MDSCs from murine bone marrow cells [21]. The mMT‐FPs and Lm‐FP were added to the culture, and the effect of the FPs on MDSC generation was assessed. Representative results from three independent experiments are shown in Fig. 5A,B. In Fig. 5A, the relative content of PMN‐MDSCs in the no‐peptide culture was 26.7%, and this proportion increased in the presence of Lm‐FP, that is, 35.1 and 38.6% at 10 nm (7.5 ng·mL^−1^) and 100 nm (75 ng·mL^−1^), respectively. In contrast, the relative content of M‐MDSCs decreased with the addition of Lm‐FP (68.5%: no peptide control, 59.0%: 10 nm Lm‐FP, 54.6%: 100 nm Lm‐FP). A similar trend was observed with mMT‐FPs. Representative results for mMT‐ND1 and mMT‐ND5 are shown in Fig. 5B. Figure 5C shows the contents of PMN‐MDSCs and M‐MDSCs for each FP group and matched no‐peptide controls in each experiment. An increase in PMN‐MDSC content due to the addition of FPs was confirmed in all five mMT‐FPs and Lm‐FP, while a reciprocal decrease in M‐MDSCs was observed with all peptides. The total number of MDSCs obtained from the 4‐day *in vitro* culture varied between experiments, and no consistent trend was observed in the yield of MDSCs (Fig. S1).

**Fig. 6:**
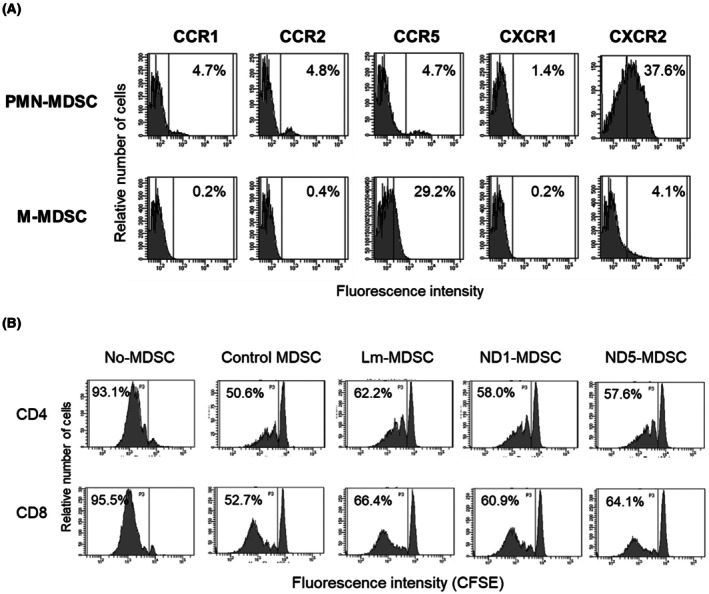
Effects of mMT‐FPs on chemokine receptor expression and immunosuppressive function of MDSCs. (A) Cell surface expression of various chemokine receptors in the generated MDSCs of no‐peptide controls. (B) Effects of mMT‐FPs on MDSC immunosuppressive function. The CFSE‐labeled spleen cells, with different cell ratios of MDSCs, were stimulated with anti‐CD3 and anti‐CD28 antibody–conjugated beads, and the subsequent cell proliferation of CD4 and CD8 cells was analyzed on day 3. Representative results for Lm‐FP, mMT‐ND1, and mMT‐ND5 at a 1 : 0.4 spleen cell/MDSC ratio. Percentages of proliferated cells following the CD3/CD28 stimulation. Representative results from the three independent experiments.

We next analyzed the effect of MT‐FPs on the surface expression of chemokine receptors in *in vitro*–generated MDSCs. The expression profiles of CCR1, CCR2, CCR5, CXCR1, and CXCR2 were evaluated in both MDSC subsets from no‐peptide control cultures in three independent experiments (Fig. 6A). CCR1 and CCR2 were partially expressed in PMN‐MDSCs (4.7 ± 0.3% and 5.0 ± 0.6%, respectively), but no expression was detected in M‐MDSCs. CCR5 was expressed in both PMN‐ and M‐MDSCs, with preferential expression in M‐MDSCs (5.2 ± 1.1% and 27.0 ± 9.9%, respectively). CXCR2 was expressed in both subsets, with higher expression in PMN‐MDSCs than in M‐MDSCs (45.4 ± 8.8% and 4.3 ± 1.0%, respectively). CXCR1 was partially expressed in PMN‐MDSCs (1.8 ± 0.4%) and was not detected in M‐MDSCs. Chemokine receptor expression patterns in the FP‐treated groups closely resembled those of the no‐peptide controls (Fig. S2). These findings suggest that mMT‐FPs do not alter chemokine receptor expression in MDSCs generated *in vitro*. We next examined whether mMT‐FPs affected the immunosuppressive function of MDSCs. Representative results at a 1 : 0.4 spleen cell/MDSC ratio from the three independent experiments are shown in Fig. 6B. MDSCs generated without FPs strongly suppressed CD4 and CD8 T‐cell proliferation; the percentages of proliferated CD4 and CD8 cells dropped from 93.1 and 95.5% (no‐MDSC control) to 50.6 and 52.7%, respectively, upon MDSC co‐culture. In contrast, MDSCs generated in the presence of FPs showed approximately 10% higher proliferation in both T‐cell subsets, indicating weaker suppressive activity compared to control MDSCs.

## Discussion

Mitochondrial DNA encodes 13 proteins, including two ATP synthases, three cytochrome c oxidases, one cytochrome b, and seven NADH dehydrogenases, all of which compose complexes involved in the oxidative phosphorylation process [22]. N‐formyl methionine is used to initiate the synthesis of these 13 proteins, from which N‐formylated peptides are generated and released into the extracellular space after cell lysis [6, 10, 11]. There are different neutrophil‐activating potentials among MT‐FPs derived from the 13 proteins; specifically, 6 of the 13 human MT‐FPs have been identified as neutrophil‐activating peptides in humans [19]. However, the potential of murine MT‐FPs (mMT‐FPs) to activate murine immune cells has not been thoroughly investigated. In the present study, we examined the neutrophil activation activity of 13 pentapeptides derived from mMT‐FPs and identified 5 as potential activators of murine bone marrow neutrophils. There is no significant difference between the five active peptides and the non‐active peptides in hydrophobicity or amino acid sequence similarity. Therefore, it is unclear why only five peptides demonstrate activity. One possible explanation is that active and non‐active peptides differ in their binding affinity to FPRs, although we have not yet determined the affinity. We further examined the effects of these five peptides on the *in vitro* generation of MDSCs from murine bone marrow cells, since our syngeneic tumor transplantation study using MTFMT‐knockout tumors suggested that MT‐FPs induce MDSCs *in vivo* [9]. However, we did not detect any consistent increase in total MDSC numbers after 4 days of culture in either the mMT‐FP or Lm‐FP group. This discrepancy with the previous *in vivo* findings may reflect the role of cellular trafficking, as MDSC recruitment into tumor tissues occurs *in vivo* [23] but is absent in the *in vitro* generation system. The dominant MDSC population in tumor tissues was PMN‐MDSCs in both the wild‐type and MTFMT‐knockout tumors. The proportion of PMN‐MDSCs increased when mMT‐FPs were intratumorally injected [9]. Consistent with these observations, the relative proportion of PMN‐MDSCs increased in the mMT‐FP–treated cultures in the present study, accompanied by reciprocal decrease in M‐MDSCs.

Crucial roles of several chemokines and their receptors in MDSC trafficking have been reported [23, 24]. We therefore examined chemokine receptor expression in the *in vitro*–generated MDSCs. However, MT‐FPs had no measurable effect on chemokine receptor expression in either subset. These findings suggest that MT‐FPs may influence chemokine production in the TME but do not modulate receptor expression in MDSCs themselves.

We further examined the immunosuppressive function of the MDSCs generated in the presence or absence of mMT‐FPs. Compared with control MDSCs, those generated with mMT‐FPs or Lm‐FP exhibited weaker suppression of CD4 and CD8 T‐cell proliferation. This finding is consistent with previous reports that M‐MDSCs are more immunosuppressive than PMN‐MDSCs [4]. The reduced immunosuppressive activity of the total MDSC population in FP‐treated groups corresponded with the decreased proportion of M‐MDSCs. These results suggested that neither the mMT‐FPs nor Lm‐FP affected the per‐cell immunosuppressive activity of the *in vitro*–generated PMN‐MDSCs and M‐MDSCs. Ligand binding to FPRs activates three predominant signaling pathways: phosphoinositide‐3 kinase (PI3K), mitogen‐activated protein kinase (MAPK), and phospholipase C (PLC). The roles of PI3K signaling in regulating the physiological processes of macrophages and neutrophils, as well as in the proliferation and survival of MDSCs, have been reported [25]. The MAPK pathway is also implicated in the expansion and differentiation of MDSC subsets [26]. For example, activation of the MAPK pathway mediated by adenosine receptor A2b modulated the PMN‐MDSC/M‐MDSC balance. The shift in MDSC subset balance was thought to result from the higher expression of A2b on PMN‐MDSCs compared with M‐MDSCs. Although the precise mechanisms underlying the FP‐mediated alteration in the PMN−/M‐MDSC balance observed in the present study remain unclear, a possible mechanism is differential FPR expression on precursor or lineage‐committed immature myeloid cells of PMN‐ and M‐MDSCs.

In conclusion, we identified five mMT‐FPs (mMT‐ATP6, mMT‐CO1, mMT‐ND1, mMT‐ND4, and mMT‐ND5) that activated neutrophils in mice. We further examined the effects of these peptides on the *in vitro* generation of MDSCs, revealing an increase in PMN‐MDSCs along with a corresponding decrease in M‐MDSCs following the addition of mMT‐FPs. These findings support the previous results obtained from *in vivo* tumor transplantation models and suggest that both MT‐FPs and enzymes involved in their metabolisms, such as peptide deformylase, may serve as new targets for cancer therapy. The effects of MT‐FPs on cancer and other diseases in animal models remain poorly understood due to limited information about MT‐FPs in experimental species. Thus, in addition to cancer research, the identification of functional mMT‐FPs may aid in the development of murine models for MT‐FP–related non‐cancerous diseases, including systemic inflammatory response syndrome resulting from trauma or cardiac surgery and acute respiratory distress syndromes [27, 28].

## Conflict of interest

The authors declare no conflict of interest.

## Author contributions

MO designed and performed the experiments, analyzed and validated data, and wrote, edited, and reviewed the manuscript. SK performed the experiments and analyzed data. AY conceptualized and supervised the study, analyzed data, and wrote, reviewed, and edited the manuscript. MO and AY confirm the authenticity of all the raw data. All authors commented on previous versions of the manuscript. All authors have read and approved the final version of the manuscript.

## Supporting information


**Fig. S1.** Absolute numbers of MDSCs after 4 days of bone marrow culture with GM‐CSF, IL‐6, and formyl peptides.

**Fig. S2.** Effect of MT‐FPs on the surface expression of chemokine receptors in *in vitro*–generated MDSCs.

## Data Availability

The data that support the findings of this study are available from the corresponding author akiymd@med.kurume-u.ac.jp upon reasonable request.
