# Supplementary figures and images for: Identification of functional murine mitochondrial formyl peptides and their effects on myeloid‐derived suppressor cell generation
Source: FEBS Open Bio. 2026 Feb 11;16(7):1387–99. doi: 10.1002/2211-5463.70209 (PMC13327073; doi:10.1002/2211-5463.70209)

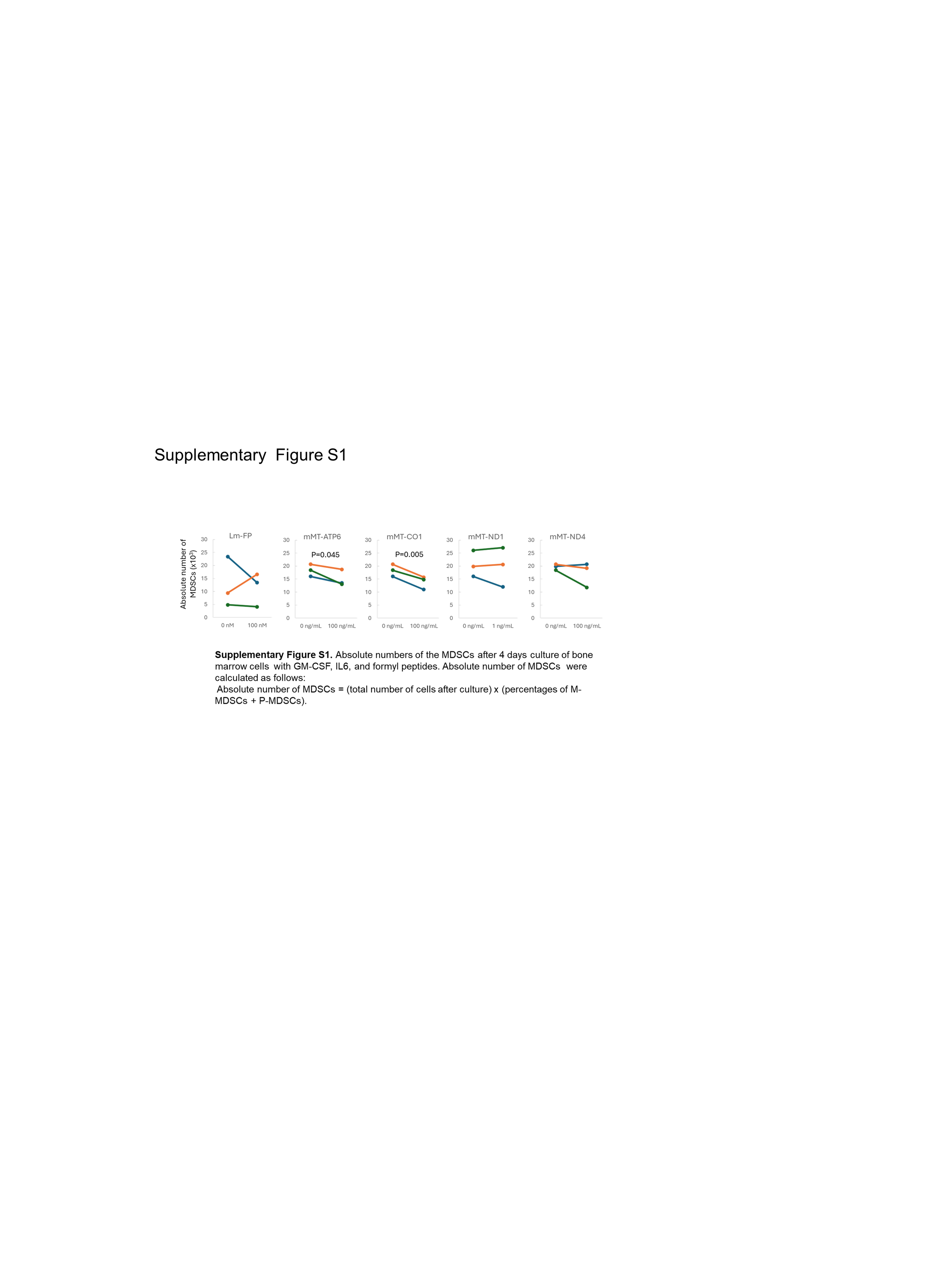

Supplement: Supplementary file 1 — Fig. S1. Absolute numbers of MDSCs after 4 days of bone marrow culture with GM‐CSF, IL‐6, and formyl peptides. [file FEB4-16-1387-s001.pdf]

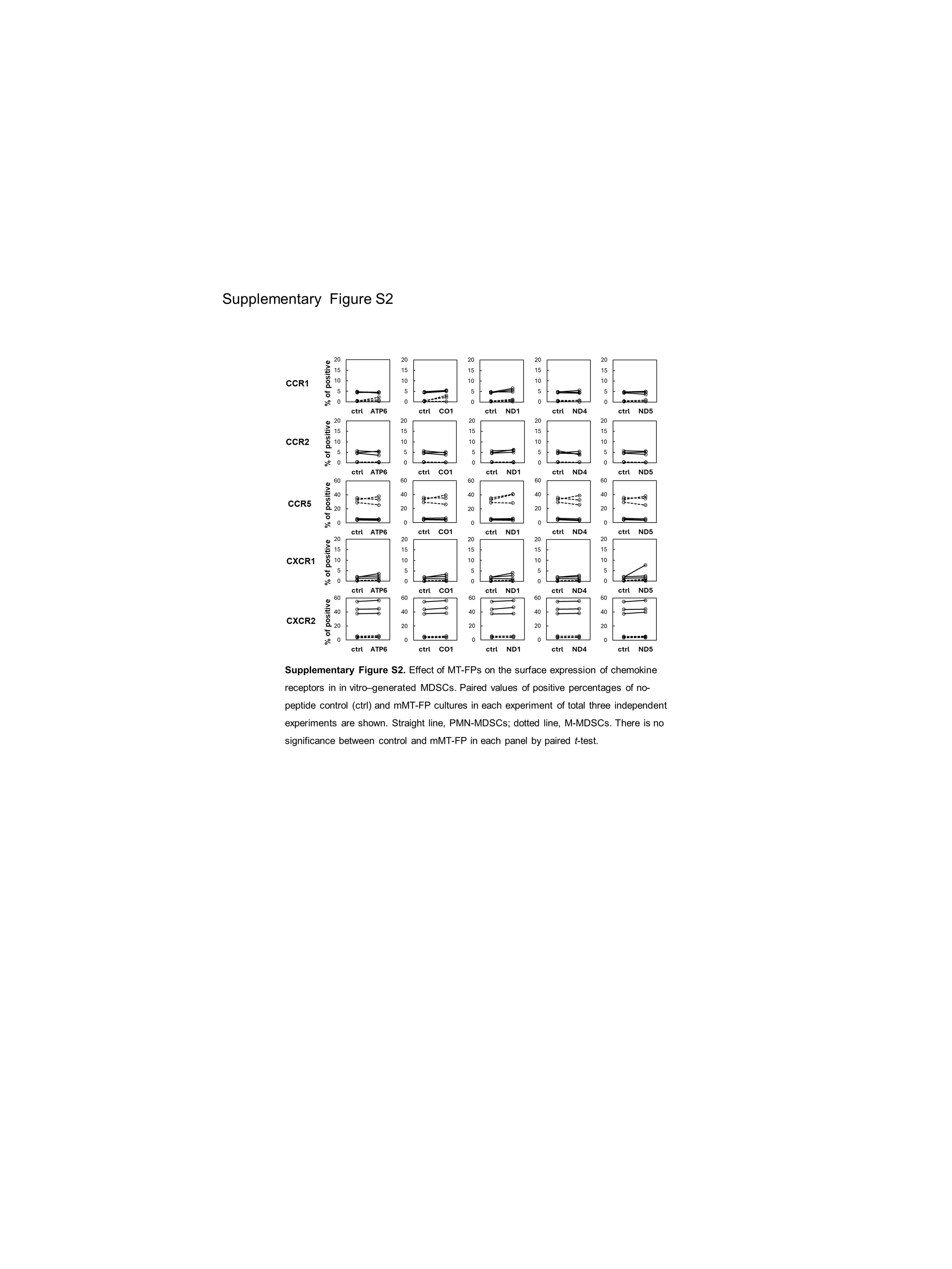

Supplement: Supplementary file 2 — Fig. S2. Effect of MT‐FPs on the surface expression of chemokine receptors in in vitro–generated MDSCs. [file FEB4-16-1387-s002.pdf]
